# Editorial: Ion channels and transporters in diabetes and metabolic diseases

**DOI:** 10.3389/fphar.2022.975812

**Published:** 2022-08-22

**Authors:** Michael Puljung, Elizabeth Haythorne, Maria Rohm, Natascia Vedovato

**Affiliations:** ^1^ Department of Chemistry and the Neuroscience Program, Trinity College, Hartford, CT, United States; ^2^ Department of Physiology, Anatomy and Genetics, University of Oxford, Oxford, United Kingdom; ^3^ Institute for Diabetes and Cancer, Helmholtz Center Munich, Neuherberg, Germany; ^4^ German Center for Diabetes Research (DZD), Neuherberg, Germany

**Keywords:** drug therapy, metabolic disease, repurposable drugs, membrane protein, aging, fat storage, hormone

Textbooks frequently divide plasma membrane receptors into two distinct camps: metabotropic receptors that initiate intracellular signaling cascades in response to extracellular messengers and ionotropic receptors that exert rapid and direct effects on membrane excitability. However, rigid, this-or-that classifications cannot fully capture the bumpy landscape of plasma membrane receptors and their effects on cell signaling. Outside of textbooks, numerous ion channels and transporters serve metabotropic functions, directly impacting chemical signaling pathways (for example, via changes in intracellular Ca^2+^) and many are, in turn, directly activated and inhibited by changes in the concentrations of cellular messengers like cyclic nucleotides, ATP/ADP, anionic phospholipids, and inositol triphosphate. This provides a direct mechanism by which changes in electrical excitability can affect cellular/systemic metabolism and vice versa. Beyond their role coordinating potential changes in the plasma membranes of excitable cells, ion channels and transporters also influence the membranes of intracellular organelles like the endoplasmic reticulum, endosomes, lysosomes and mitochondria.

Channels and transporters set cytoplasmic and vesicular ion concentrations and pH, impact transepithelial transport, regulate cell volume, and control hormone secretion. Not surprisingly, their dysfunction impacts numerous metabolic diseases ranging from hypertension to kidney stones. The pathology and treatment of diabetes, a disease of impaired glucose homeostasis and aberrant insulin secretion, are impacted by numerous channels and transporters. The number of individuals with diabetes and metabolic diseases worldwide has risen exponentially over the last 40 years owing in part to an aging world population and epidemic rise in obesity rates. Therefore, there is a pressing need to develop more effective therapies to improve patient outcomes.

We are happy to present a collection of articles in *Frontiers in Pharmacology* highlighting ion channels and membrane transporters as new and repurposed drug targets for the treatment of metabolic disorders. The article by Cyranka et al. and review by Armour et al. identify two overlooked candidates that may help restore normal hormone secretion in diabetics. Cyranka et al. used GLUTag cells (a convenient model for enteroendocrine L-cells) to demonstrate that inhibition of NMDA-type glutamate receptors enhances secretion of the incretin hormone GLP-1, which in turn promotes insulin secretion. Armour et al. present evidence that hyperglycemia promotes Na^+^ entry into pancreatic α-cells via the Na^+^-coupled glucose transporter SGLT1. The increase in Na^+^ lowers intracellular pH and disrupts energy balance, affecting normal glucagon secretion.

Closure of pancreatic ATP-sensitive K^+^ (K_ATP_) channels triggers insulin secretion from pancreatic β-cells. K_ATP_ inhibiting drugs like sulfonylureas and glinides have been used for decades to treat type 2 diabetes and neonatal diabetes (ND) caused by overactive K_ATP_ channels. Unfortunately, some ND mutations are so severe that the usual drug treatments offer no relief. Furthermore, Cantú syndrome, associated with cardiovascular problems, skeletal malformations and hypertrichosis, is caused by mutations in a K_ATP_ channel subtype that is not strongly inhibited by sulfonylureas. With this in mind, Chen et al. used extensive molecular dynamics simulations and pharmacophore modeling to identify three drugs (all in current clinical use) that inhibit K_ATP_ channels by a direct interaction with the channel’s pore. Crucially, these drugs inhibit K_ATP_ channels bearing mutations similar to those that cause Cantú syndrome, offering some promise for the treatment of drug-resistant K_ATP_ channelopathies.

Finally, we offer papers from Chiang et al. and García-Casas et al. that address two of the most prevalent risk factors for metabolic disorders: obesity and aging. Chiang et al. used 3T3-L1 cells to investigate the influence of P2X7 receptor activation on lipid accumulation during differentiation in adipocytes. García-Casas et al. investigated the mechanism by which the Na^+^/Ca^2+^ exchanger inhibitor CGP37157 can extend the healthy lifespan of *C. elegans*.

The work presented here showcases only a sliver of the myriad connections between ion transport and metabolic disease. However, we hope that the new and reconsidered therapies and therapeutic targets presented in this Research Topic will provoke connections, collaborations, and discoveries that may impact the millions suffering from metabolic diseases.

